# Streptococcus pyogenes Capsule Promotes Microcolony-Independent Biofilm Formation

**DOI:** 10.1128/JB.00052-19

**Published:** 2019-08-22

**Authors:** Artur Matysik, Kimberly A. Kline

**Affiliations:** a Singapore Centre for Environmental Life Sciences Engineering, Nanyang Technological University, Singapore, Singapore; b School of Biological Sciences, Nanyang Technological University, Singapore, Singapore; Geisel School of Medicine at Dartmouth

**Keywords:** group A streptococcus, *Streptococcus pyogenes*, adherence, biofilms, capsule, static biofilm assay

## Abstract

Biofilms play an important role in the pathogenesis of group A streptococcus (GAS), a Gram-positive pathogen responsible for a wide range of infections and with a significant public health impact. Although most GAS serotypes are able to form biofilms, there is a large amount of heterogeneity between individual strains in biofilm formation, as measured by standard crystal violet assays. It is generally accepted that biofilm formation includes the initial adhesion of bacterial cells to a surface followed by microcolony formation, biofilm maturation, and extensive production of extracellular matrix that links together proliferating cells and provides a scaffold for the three-dimensional (3D) biofilm structure. However, our studies show that for GAS strain JS95, microcolony formation is not an essential step in static biofilm formation, and instead, biofilm can be effectively formed from slow-growing or nonreplicating late-exponential- or early-stationary-phase planktonic cells via sedimentation and fixation of GAS chains. In addition, we show that the GAS capsule specifically contributes to the alternative sedimentation-initiated biofilms. Microcolony-independent sedimentation biofilms are similar in morphology and 3D structure to biofilms initiated by actively dividing planktonic bacteria. We conclude that GAS can form biofilms by an alternate noncanonical mechanism that does not require transition from microcolony formation to biofilm maturation and which may be obscured by biofilm phenotypes that arise via the classical biofilm maturation processes.

**IMPORTANCE** The static biofilm assay is a common tool for easy biomass quantification of biofilm-forming bacteria. However, Streptococcus pyogenes biofilm formation as measured by the static assay is strain dependent and yields heterogeneous results for different strains of the same serotype. In this study, we show that two independent mechanisms, for which the protective capsule contributes opposing functions, may contribute to static biofilm formation. We propose that separation of these mechanisms for biofilm formation might uncover previously unappreciated biofilm phenotypes that may otherwise be masked in the classic static assay.

## Abstract (precis)

The static biofilm assay is a common tool for easy biomass quantification of biofilm-forming bacteria. However, Streptococcus pyogenes biofilm formation as measured by the static assay is strain dependent and yields heterogeneous results for different strains of the same serotype. In this study, we show that two independent mechanisms, for which the protective capsule contributes opposing functions, may contribute to static biofilm formation. We propose that separation of these mechanisms for biofilm formation might uncover previously unappreciated biofilm phenotypes that may otherwise be masked in the classic static assay.

## INTRODUCTION

Streptococcus pyogenes (group A streptococcus [GAS]) is a human pathogen responsible for a variety of disease states, ranging from superficial infections such as pharyngitis to severe infections such as necrotizing fasciitis, with a global impact on mortality and morbidity (1). Biofilm formation is thought to be an important GAS virulence factor, and many GAS strains can form biofilms *in vitro* (1–3). Biofilm-like GAS communities have been observed in tonsillar reticulated crypts, suggesting a role for biofilm in asymptomatic GAS carriage (4). Increased antibiotic tolerance of GAS biofilms has been also proposed as an important reason for antibiotic treatment failure (2, 5). However, the ability to form biofilm is often strain dependent and can be heterogeneous, even for isolates belonging to the same serotype. Differential regulation of primary adhesin expression, aggregation tendency, or yet unknown alternative mechanisms responsible for biofilm maturation have been proposed to explain this variability (1, 3).

Static and flow cell biofilm assays are the two most commonly used methods to study biofilm development in GAS and other organisms. Flow cell biofilm assays have the advantage of biofilm growth in the absence of planktonic cells in a nutrient-rich metabolite-poor environment, enabling continuous and detailed observation of biofilm maturation over time. However, flow cell biofilm assays are labor intensive to assemble and perform, limiting the variety of conditions that can be screened in a quantitative way (6, 7). In contrast, the assay for biofilm growth under static conditions, typically monitored in 24-well polystyrene plates followed by crystal violet or safranin staining, is a simple high-throughput method to quantify overall biofilm biomass. A drawback of the static biofilm assay is nutrient depletion and metabolite accumulation over time, both of which can affect biofilm maturation (8). Static biofilm assays are strongly influenced by primary cell-to-surface interactions, rendering analysis of the entire biofilm life cycle more difficult (8, 9). Despite these limitations, static biofilm assays remain the major tool for quantitative assessment of GAS biofilm formation (3, 8, 10–15).

Several recent studies by us and others have implicated biofilm formation in necrotizing fasciitis (NF) (16–19). In this study, we sought to dissect the mechanism of biofilm formation of the GAS NF-associated strain JS95. Modifying the commonly used static biofilm assay, we discovered that two parallel mechanisms contribute to GAS biofilm formation: one that proceeds through a classic microcolony proliferation-dependent stage and another that is seeded via proliferation-independent sedimentation. Capsule has been reported as a GAS biofilm factor, but its precise contribution to biofilm development has been unclear (1, 13). Using the modified static biofilm assay developed here, we demonstrate opposing contributions of the capsule in each biofilm development mechanism.

## RESULTS

### GAS JS95 biofilm *in vitro*.

**FIG 1:**
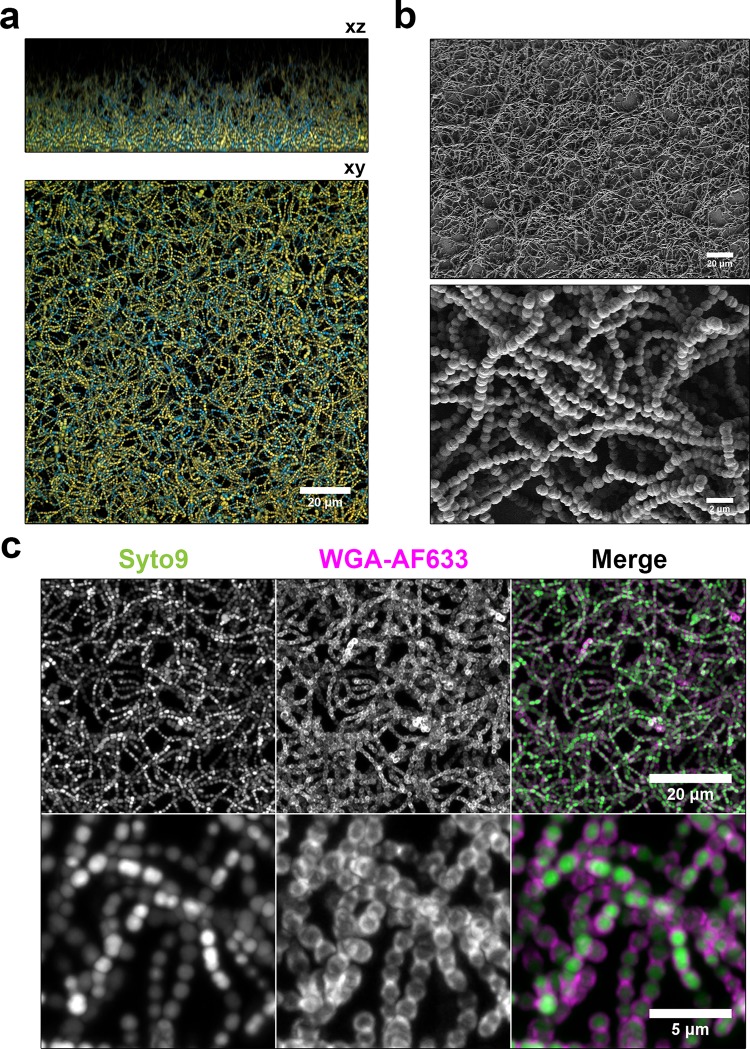
GAS JS95 biofilm morphology. (a) JS95 biofilm was grown on polystyrene plate stained with Hoechst 33342 (blue), Syto9 (green), and propidium iodide (red). Orange color represents dead cells. A representative color-merged volume projection of a Z-stack is shown. (b) Scanning electron micrographs of JS95 biofilm grown on polystyrene at ×500 (top) and ×5,000 (bottom) magnification. (c) Maximum projection of JS95 biofilm Z-stacks stained with Syto9 (dsDNA) and WGA-Alexa Fluor 633 (carbohydrate/EPS).

We previously described determinants that are important for necrotizing GAS strain JS95 (M14 serotype) biofilm formation in association with host cells; we therefore grew GAS in Dulbecco's modified Eagle's medium (DMEM) cell culture medium in that study (17). Here, we used the more commonly used GAS growth medium, Todd-Hewitt broth with 2% yeast extract (THY) supplemented with 0.5% glucose, which was previously demonstrated to improve adherence and biofilm formation (1, 13, 15, 20). Biofilms were assayed in 24-well polystyrene plates following static incubation at 37°C for 24 h. Although some GAS strains require additional surface coating (e.g., poly-l-lysine, collagen, fibronectin, and fibrinogen) for GAS attachment and biofilm formation (3, 13), JS95 formed biofilm on polystyrene plates without additional surface treatment. Confocal microscopy showed an extensive three-dimensional biofilm structure (Fig. 1a), which is typical of most GAS biofilms (3). As expected, we also observed a similar biofilm architecture by scanning electron microscopy (SEM) (Fig. 1b). Although biofilm matrix-like structures have been observed by SEM in some GAS biofilm studies (3, 21), we did not observe similar structures. An absence of visible extracellular polymeric substance (EPS) matrix in SEM images of GAS biofilms was previously reported for serotypes M6, M18, and M49 (3), whereas matrix-like material has been observed in MGAS5005 biofilms (21). The heterogeneity in visible biofilm matrices may be due to differences in sample preparation, strains differences, or other experimental variations. Wheat-germ agglutinin (WGA), a lectin which binds carbohydrate-containing extracellular polymeric substances (EPS), has been used as a marker of biofilm matrix for GAS and other organisms (16, 22, 23); however, WGA staining did not reveal extracellular biofilm matrix for strain JS95 (Fig. 1c). We also confirmed that the apparent cell-associated WGA staining is not due to capsule staining by WGA as reported in some studies (16), since the *hasA* capsule mutant showed an identical staining pattern (see Fig. S1 in the supplemental material). Hence, these findings show that GAS strain JS95 forms the three-dimensional (3D) structures consistent with biofilm architecture described for other GAS strains, but we were unable to visualize extracellular biofilm matrix.

### GAS can form biofilms from early-stationary-phase planktonic culture.

**FIG 2:**
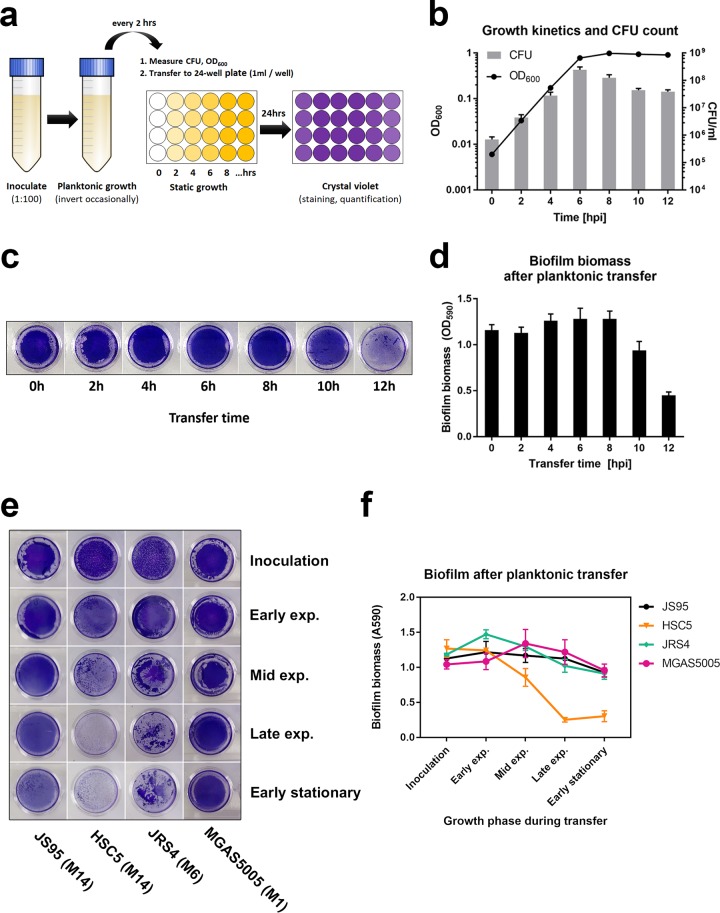
Planktonic transfer assay. (a) Assay scheme. Cells grown in planktonic culture in a 50-ml conical tube were measured for CFU and OD_600_ and then transferred to a separate 24-well plate. After a 24-h incubation, biofilm biomass was measured by crystal violet (CV) staining. (b) OD_600_ and CFU counts at the indicated time points during planktonic growth. (c) Images of biofilm stained with CV. (d) Biofilm biomass quantification by OD_590_ measurement of solubilized crystal violet. Images (e) and quantification (f) of biofilm formed by JS95, HSC5, JRS4, and MGAS5005 strains after planktonic transfer at various growth phases. Graphs show mean values ± standard deviations.

**FIG 3:**
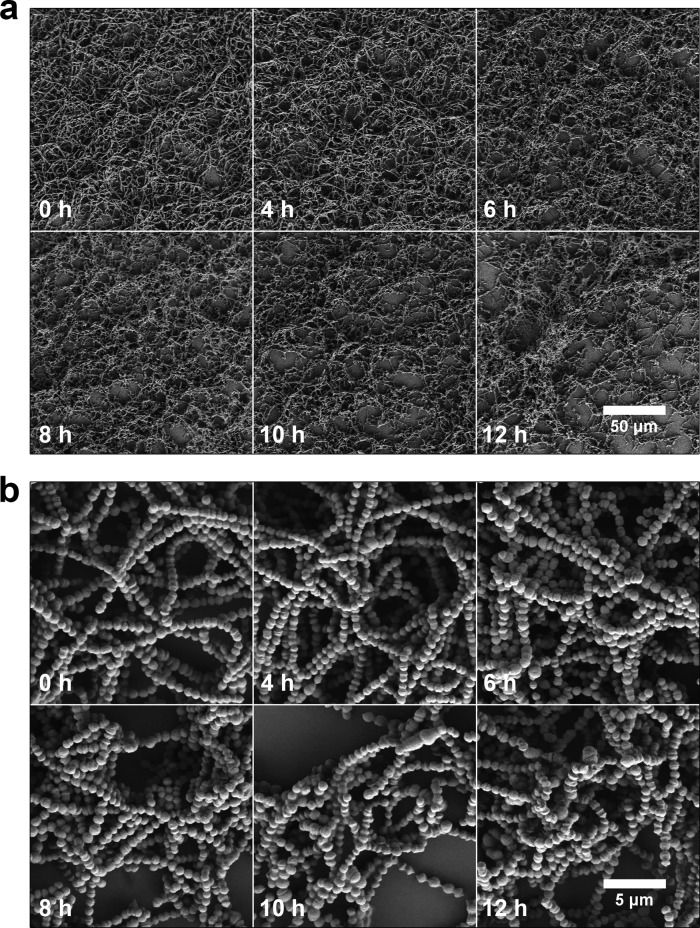
SEM images of GAS JS95 biofilm after planktonic transfer. Scanning electron micrographs (SEM) of biofilms from transferred planktonic cultures at the indicated time points. Biofilms were grown in 6-well polystyrene plates and imaged at ×500 (a) and ×5,000 (b) magnification.

Biofilm development is generally thought to be initiated by cell adhesion to a surface followed by the formation of microcolonies, extensive extracellular matrix production, and proliferation to result in a mature biofilm structure (1, 9, 24). Typically, GAS static biofilm assays are conducted by dilution of overnight cultures into fresh growth medium and incubation in 24-well polystyrene plates without agitation for several hours prior to washing and staining for biofilm biomass (8, 13, 16, 20, 25–27). Several GAS adhesins, such as M-protein, collagen-like surface protein, and fibronectin-binding protein, are upregulated in early stages of planktonic growth (28) and are thought to be important for static biofilm formation (3). We therefore examined biofilm formation at different stages of planktonic growth, predicting that biofilms initiated from later stages of planktonic growth would exhibit attenuated biofilm formation due to reduced adhesin expression (28) and reduced proliferation. To test this hypothesis, we designed a simple planktonic transfer assay, in which the same volumes of planktonic GAS culture grown in a conical tube (with occasional agitation to prevent biofilm formation and sedimentation) were transferred to a 24-well plate (1 ml/well) at various times throughout the growth curve. Therefore, although fewer cells were transferred at early time points, the medium contained nutrients allowing for continued growth and biofilm formation. In contrast, at later time points, despite a high number of cells being transferred, the medium lacked nutrients supporting further growth. We measured the optical density at 600 nm (OD_600_) and determined the CFU at each time point to monitor the growth rate and planktonic cell viability prior to transfer. Twenty-four-well plates were then incubated for 24 h, and the resulting biofilm biomass was quantified using crystal violet (CV) (Fig. 2a). Based on this analysis, biofilms were initiated from planktonic cultures at time points approximately representing the following stages of growth: inoculation (0 h), early exponential (2 h), mid exponential (4 h), late exponential (6 h), early stationary (8 h), and later stationary (10 and 12 h). CFU values enumerated at the time of transfer peaked at 6 h (2.4 × 10^8^ CFU/ml) and then decreased to 4 × 10^7^ CFU/ml at the last two time points (10 and 12 h) (Fig. 2b). Surprisingly, biofilm formation ability remained unchanged until later stationary phase (10 h), when biofilm biomass dropped by 25% compared to that at earlier time points (Fig. 2c and d). In other words, a substantial amount of biofilm biomass was still formed from planktonic culture in early stationary phase (8 h), when cellular proliferation is presumably diminishing, and the ability to form biofilm was lost only at 12 h postinfection. We also tested strains MGAS5005 (serotype M1) and JRS4 (serotype M6) and, despite visible differences in biofilm morphology, observed that both exhibited a strong biofilm phenotype when transferred at later stages of growth. In contrast, strain HSC5 (serotype M14) did not form robust biofilm after late-exponential-phase transfer (Fig. 2e and f). We next investigated the microphenotype of biofilms initiated from cultures at each growth phase. Consistent with CV assay results (Fig. 2c and d), SEM images showed slightly decreased surface coverage for biofilms initiated from 10- and 12-h time points. In addition, the overall structure of biofilm initiated from 12-h stationary-phase cultures appeared less homogeneous and tended to aggregate (Fig. 3a). However, there were no major differences in biofilm structure or GAS chain organization between these biofilms (Fig. 3b). As determined by OD_600_, CFU and biomass quantification, and imaging, these findings suggest that cell proliferation itself may not be crucial for biofilm formation in this static assay. Finally, we tested whether classic and transferred biofilms differ in antibiotic tolerance, a commonly used hallmark of biofilm (1, 5, 23). We first determined the MIC and minimum bactericidal concentration (MBC) for planktonic cultures and then compared the minimum biofilm eradication concentrations (MBECs) for GAS JS95 biofilm upon exposure to penicillin. We observed that the MBECs of both types of biofilm were similar (differing by one double dilution of the antibiotic) and far exceeded planktonic MIC and MBC values (see Fig. S2).

### Cell proliferation is not necessary for adherent biomass accumulation.

**FIG 4:**
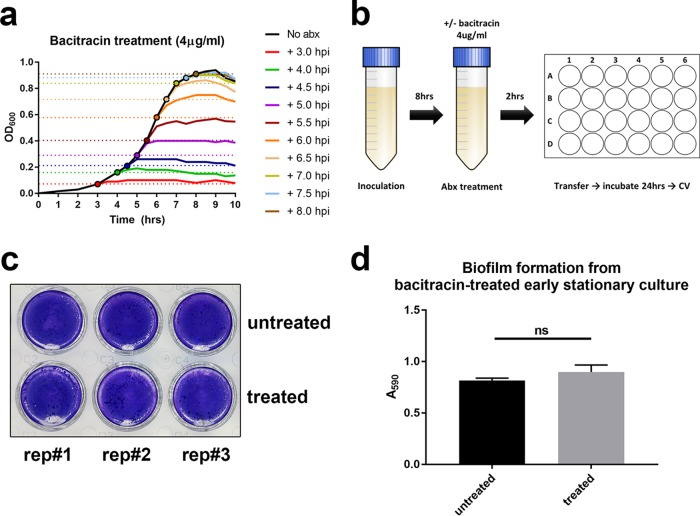
Bacitracin treated planktonic transfer assay. (a) Planktonic cultures were exposed to bacitracin (4 μg/ml) at the time points indicated by the color-matched circles, and growth inhibition was quantified by OD_600_ measurement. (b) Bacitracin treatment for planktonic transfer assay: planktonic culture was grown for 8 h (to early stationary phase) prior to bacitracin treatment. After a 2-h incubation with antibiotic, cells from the nondividing culture were transferred for static biofilm growth. (c and d) Biofilms were seeded from bacitracin treated and nontreated early stationary cells, and biomass was quantified by CV staining crystal violet after 24 h of incubation.

To determine whether cellular proliferation was indeed necessary for biofilm biomass accumulation, we treated GAS with the bacteriostatic antibiotic bacitracin. We first confirmed that bacitracin inhibits GAS proliferation at 4 μg/ml, as reported previously (29). Independent of the time of bacitracin addition (3, 4, 4.5, 5, 5.5, 6, 6.6, 7, 7.5, and 8 h postinoculation), treatment with 4 μg/ml bacitracin resulted in an OD_600_ plateau within approximately 1 h, with no more than a 30% increase in turbidity after the time of treatment (Fig. 4a). Therefore, we reasoned that after 2 h of bacitracin treatment, there would be no further proliferation. Hence, we repeated the planktonic transfer assay, growing the planktonic culture for 8 h (early stationary phase) and adding bacitracin at 4 μg/ml for an additional 2 h of incubation and then transferring the nonproliferating planktonic cells to 24-well plate for static biofilm assay (Fig. 4b). Crystal violet staining showed equally strong biofilm biomasses regardless of bacitracin treatment (Fig. 4c and d). Together, these observations suggest that proliferation is not essential for biofilm biomass accumulation, which can arise from cells which are no longer dividing.

### Planktonic transfer assay uncovers capsule-dependent phenotype differences, masked in the classic CV assay.

**FIG 5:**
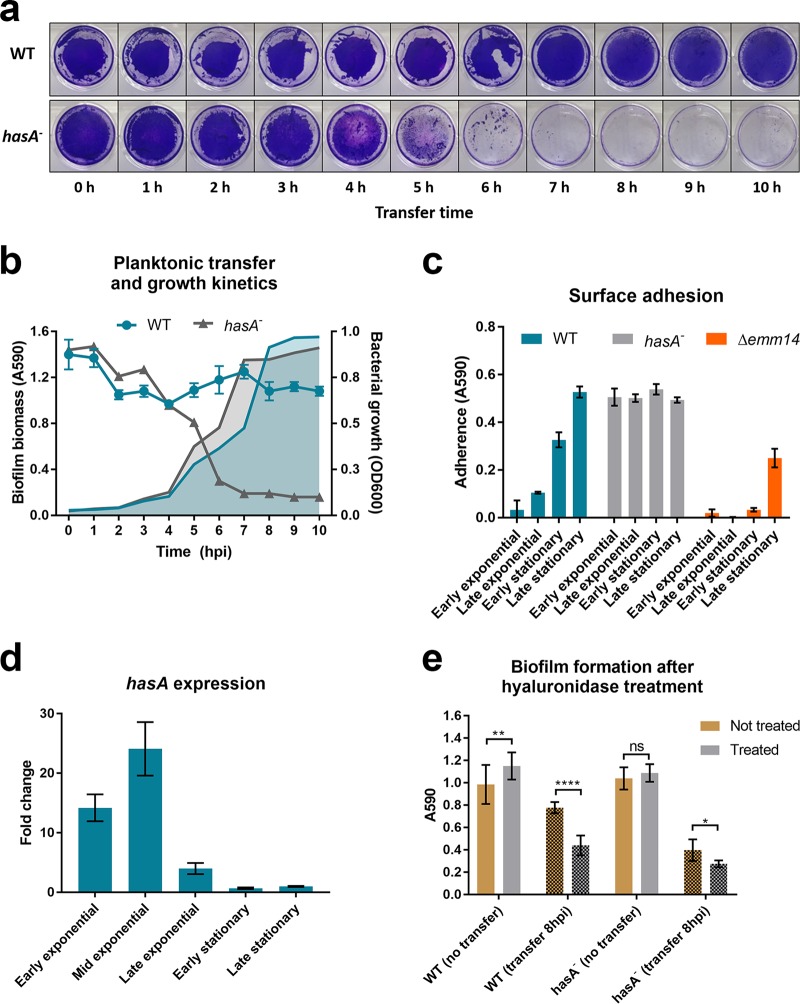
Planktonic transfer assay with capsule deficient GAS mutant. (a) CV stained WT and *hasA* mutant biofilms transferred at the indicated time points. (b) Planktonic bacterial growth (OD_600_) and biofilm biomass quantified by CV staining (*A*_590_) at the time of transfer. (c) Adherence of JS95 planktonic cultures (normalized to the same OD_600_) to polystyrene, derived from the indicated growth phases. (d) *hasA* expression at different phases of growth in THY plus 0.5% glucose relative to that in late-stationary-phase (15 hpi) culture. (e) Hyaluronidase treatment (50 μg/ml) of classic biofilms transferred at 8 h (early stationary phase). Graphs show mean values ± standard deviations.

Cho and Caparon previously showed that a GAS mutant deficient in capsule production was able to form biofilm under static conditions as well as the isogenic parental wild-type strain but was unable to develop biofilm under flow biofilm conditions (19). We therefore hypothesized that some of the phenotypic inconsistencies between these assays could be dissected using the planktonic transfer assay established in this study. We performed planktonic transfer assays at 1-h intervals for a total of 10 h, comparing wild-type JS95 and an isogenic *hasA* mutant deficient for capsule production (17) (Fig. 5a). The wild-type (WT) GAS JS95 biofilms inoculated from all growth phases, including early stationary phase, resulted in dense biomass accumulation, as was observed as described above (Fig. 2c and d). In addition, we observed visually distinct CV staining for early transfer times, whereby the *hasA* mutant biofilm appeared more robust, although CV quantification did not reveal significant differences (Fig. 5a and b). In contrast, the *hasA* mutant was unable to accumulate biomass when biofilms were initiated from cultures at mid-exponential phase onward (Fig. 5a and b). Interestingly, both strains (WT and *hasA* mutant) were unable to form biofilm in a Calgary biofilm device (CBD), in which sedimentation of cells that might contribute to biofilm formation is excluded due to the inverted device geometry (see Fig. S3) (7, 30, 31). A strong biofilm was, however, observed in Pseudomonas aeruginosa which served as a positive control (9). To explore the contribution of adhesins to biofilm formation by each mechanism, we used a simple adhesion assay in which planktonic cultures at different growth stages were normalized to the same optical density and incubated in a 24-well plate for 30 min at 37°C, the nonadherent were cells removed by washing, and the adherent cells were stained using crystal violet. We observed that adhesion of the capsule mutant was similar at all tested growth phases (Fig. 5c). Adherence of the wild-type strain increased gradually from low levels in early exponential growth to late stationary phase where it reached the same level as the capsule mutant. We confirmed that, as in other GAS strains, *hasA* expression peaked at mid-exponential growth and then decreased rapidly (Fig. 5d) (32, 33). Together with the fact that capsule shedding is associated with cessation of its synthesis (34), these data provide further evidence that the capsule hinders adhesin-mediated surface attachment. In the same adhesion assay, we tested a JS95 mutant unable to produce M-protein, which is a well-studied GAS virulence factor also shown to be crucial for biofilm formation (19). In contrast, a mutant for the M-protein was nonadhesive until the late stationary phase (overnight culture), when adherence reached approximately one-half the level of that for the WT at the same growth phase, showing that surface adhesion is not solely dependent on M-protein. Finally, we tested whether exogenous hyaluronidase treatment of WT biofilms could phenocopy the *hasA* mutant biofilms. Indeed, capsule removal by hyaluronidase slightly enhanced the classic WT biofilm formation and significantly reduced biofilm formed from cultures transferred at 8 h postinoculation (hpi) (Fig. 5e). At the same time, hyaluronidase presence had little to no effect on the *hasA* mutant. These data suggest that the capsule contributes differently to biofilms depending on growth stage: it inhibits biofilm formation during early exponential growth by masking surface adhesins but promotes biofilm formation when it is initiated from later phase cultures.

## DISCUSSION

In this study, we investigated biofilm formation by the clinical GAS strain JS95 (M14 serotype), isolated from an NF patient (18). We confirmed that it forms biofilm under static conditions, showing dense three-dimensional biofilm structures of chaining cocci characteristic of many of the GAS strains. Although GAS has been shown to produce a biofilm matrix of extracellular polymeric substances (EPS), composed primarily of l-glucose and d-mannose (35), we were unable to observe an extracellular EPS matrix using microscopy for either WT or *hasA* mutant stains.

**FIG 6:**
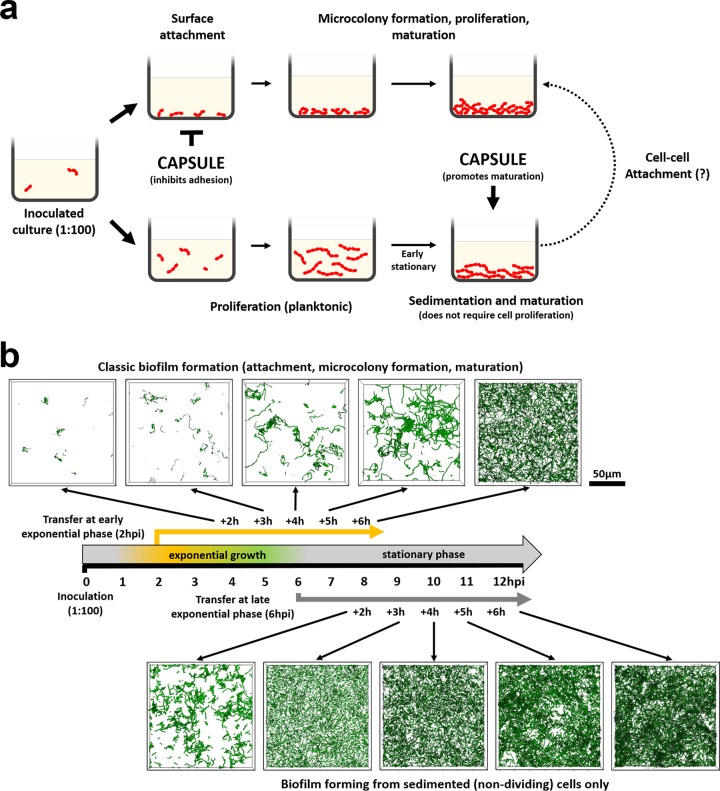
Model for static GAS biofilm formation. (a) In the classic mechanism for biofilm formation, surface attachment is followed by microcolony formation, cell proliferation, EPS production, and biofilm maturation. In the alternate pathway for biofilm formation, planktonic cells sediment when they reach a critical cell density, attach to a surface, and become fixed into biofilm structure. Both mechanisms are likely occur in the widely used static biofilm assay. (b) Time course showing biofilm development from culture transferred at early and late exponential phases. Images of biofilm developing from early-exponential-phase culture show typical steps of initial attachment and microcolony formation; transfer of late-exponential-phase cells results in moderate initial attachment (2 h of static incubation), followed by rapid increase in biofilm formation at later time points, supporting the proposed alternate sedimentation mechanism of biofilm initiation. CLSM images of Syto9-stained biofilms are rendered as volume projections.

Strain-associated differences in GAS biofilm formation have been reported. To address whether strain-independent factors might contribute to biofilm formation, we were motivated to step back and more carefully assess how GAS strain JS95 biofilm is formed using the common static biofilm assay. Using the planktonic transfer assay described here, we observed that GAS cells from planktonic cultures ranging from early exponential phase to early stationary phase can seed robust biofilm formation and biomass accumulation. We observed reduced biomass accumulation only when we seeded biofilms with cells from 12-h late-stationary-phase cultures. This phenotype was not strain specific, because GAS strains of different serotypes (JRS4 and MGAS5005) displayed a similar ability to form biofilm from late-exponential/early-stationary-phase planktonic cultures. Moreover, bacitracin inhibition of cell proliferation did not prevent biomass accumulation under these conditions. Together, these data suggested the existence of two parallel mechanisms of static biofilm formation. (i) In the first “classic” mechanism, a variety of adhesins, including M and M-like proteins, facilitate initial surface attachment, followed by microcolony formation, cell proliferation, biofilm matrix production, and biofilm maturation (9, 36). (ii) In an alternative mechanism, planktonically growing cells eventually sediment and attach to the surface in a process that is enhanced by GAS capsule, leading to biofilm formation (Fig. 6a). In support of this model, biofilm development from cells seeded from early-exponential-phase culture (2 h postinoculation) showed typical steps of cell attachment to surface (2 to 3 h posttransfer), microcolony formation (4 to 5 h posttransfer), and dense biofilm formed as early as 6 h posttransfer (8 h postinoculation). In contrast, cells seeded from late-exponential-phase culture (6 h postinoculation) showed some level of initial surface attachment 2 h posttransfer and a rapid increase of biofilm density at later time points (Fig. 6b). Both situations led to stable and dense GAS JS95 biofilm formation. Importantly, the second alternate mechanism for biofilm formation could only be uncovered using the planktonic transfer assay, where sedimentation of later-growth-phase cultures can promote biofilm biomass accumulation. In contrast, biofilm biomass accumulation in the classic static assay is likely a result of both mechanisms (early attachment and proliferation of microcolonies as well as sedimentation of later-growth-phase bacteria). A similar suggestion that sedimented cells could contribute to biofilm biomass led to the development of the Calgary biofilm device (CBD) and eliminated the possibility of sedimentation (7, 30, 31). However, GAS JS95 did not form biofilms on CBD, further suggesting a strong contribution of the alternate mechanism of GAS biofilm formation.

Although many studies have demonstrated a strong link between capsule production and GAS virulence, the role for capsule in GAS biofilm formation has been unclear (36–38). Capsule production is highly regulated during GAS growth, with minimal expression during stationary phase (enabling initial adherence in a classic biofilm model) and peak expression during exponential phase (supporting an alternate capsule-dependent model for biofilm formation) (32–34), and the same pattern can be observed in the JS95 strain. Cleary et al. noted that encapsulated cells grew in a highly aggregated state that can be disrupted by hyaluronidase treatment (39). These capsule-associated aggregates were originally described as a protective mechanism against oxidative stress, equally consistent with the protective and environmental stress-tolerant state associated with biofilms. Šmitran et al. demonstrated that enzymatic removal of capsule prior to biofilm initiation improved static biofilm formation by most GAS isolates (40), suggesting that the capsule masks biofilm-associated surface adhesins, as has been demonstrated for M-protein-mediated GAS attachment to keratinocytes (41). In the context of our revised model for GAS biofilm formation, initial capsule removal would be expected to promote classic biofilm formation, where initial adhesion is essential for biofilm development. Consistent with this, we observed altered early stage biofilm formation whereby the *hasA* mutant biofilm appeared more robust, although this was not quantifiable by CV staining. The importance of capsule in later stages of biofilm formation has been shown by Cho and Caparon, who demonstrated that a capsule mutant was unaffected in surface adhesion but was unable to form biofilm in a flow cell (19). However, both WT and the capsule mutant were fully capable of forming biofilm in the classic static biofilm assay (19). The ability of the planktonic transfer assay to detect different mechanisms of biofilm initiation enabled us to further dissect the contribution of capsule to biofilm formation. Our finding that capsule mutants are attenuated only for sedimentation-mediated microcolony-independent static biofilms may suggest that GAS sedimentation characteristics are important in the maturation of flow cell GAS biofilms. Collectively, these findings suggest that capsule may limit initial surface adherence and promote sedimentation-mediated biofilm maturation. The ability of the planktonic transfer assay to dissect the contribution of growth-stage-specific factors may be widely applicable to many other GAS biofilm factors as well.

In summary, we showed that different mechanisms contribute to static GAS biofilm formation: (i) classic surface adhesion followed by microcolony formation and biofilm maturation and (ii) sedimentation and attachment of dense planktonic bacteria to a surface followed by biofilm maturation. We showed that capsule may differentially contribute to each of these mechanisms. Separation of these two mechanisms might help to uncover phenotypes otherwise masked in static biofilm assays, enabling a better understanding of the mechanisms of GAS biofilm formation, and ultimately may inform how biofilm relates to GAS virulence.

## MATERIALS AND METHODS

### Bacterial culture, planktonic transfer assay, and biofilm assay.

GAS strain JS95, an M14 serotype isolate from an NF patient (18), a *hasA* mutant in the JS95 background, and strains JRS4 (M6 serotype), HSC5 (M14 serotype), and MGAS5005 (M1 serotype) were grown at 37°C overnight (16 h) in Todd-Hewitt liquid medium (Sigma-Aldrich) supplemented with 0.2% yeast extract (Becton, Dickinson) (THY) prior to all assays. Overnight cultures were inoculated 1:100 in fresh THY medium supplemented with 0.5% glucose in a 50-ml tube and incubated at 37°C, with 5% CO_2_ and a loose tube cap. Cultures were mixed by inverting the tube three times at 30-min intervals to prevent sedimentation. At the desired time points, 1 or 3 ml of planktonic culture was transferred to a well of a 24-well (for crystal violet staining) or 6-well (for SEM) polystyrene plate (Corning, Corning, NY), respectively. For confocal laser scanning microscopy (CLSM), 3 ml of culture was transferred to a 35-mm Ibidi imaging dish (Ibidi, Munich, Germany), which allows high-resolution CLSM imaging. Although some differences between substrates might influence biofilm formation, we did not observe a significant impact on biofilm formation between polystyrene and the Ibidi surface. Plates or Ibidi dishes were then incubated without agitation for 24 h at 37°C with 5% CO_2_. At the time of transfer, the optical density at 600 nm was measured in a 1-cm cuvette by a UVmini-1240 UV-visible (UV-Vis) spectrophotometer (Shimadzu, Japan), and bacterial viability was quantified by serial CFU enumeration. Prior to CFU plating, cultures were centrifuged for 2 min at 15,000 rcf using a tabletop 5424 centrifuge (Eppendorf, Germany) to disrupt chains (confirmed by bright-field microscopy). To inhibit bacterial proliferation, bacitracin (Sigma-Aldrich) was added at 4 μg/ml for the times indicated in the text and figures.

### Biofilm quantification.

After 24 h of growth, nonadherent cells were washed gently with phosphate-buffered saline (PBS). Biofilms were stained with 0.1% crystal violet (CV) for 15 min, and the excess CV was removed by a subsequent PBS wash. Stained biofilms were first imaged using a Mate 20 Pro camera (Huawei, China) and dissolved in 96% ethanol. Prior to OD measurement, samples were diluted to ensure a linear reading range. Absorbance was measured at 590 nm using an M200 spectrophotometer (Tecan AG, Männedorf, Switzerland).

### MIC, MBC, and MBEC assays.

Standard protocols with slight modifications were used to determine the MIC, MBC, and MBEC values (42, 43). Briefly, to determine the MIC, subcultures (inoculated 1:100) were grown overnight in a 96-well plate in THY with serially diluted antibiotic. Turbidity was assessed using a plate reader (Tecan AG, Männedorf, Switzerland) at 600 nm to determine the minimal antibiotic concentration that inhibited bacterial growth. Cultures were then subcultured onto THY agar plates to determine the MBC. To determine the MBEC, biofilms were prepared by inoculating THY plus 0.5% glucose 1:100 from an overnight culture and transferring (1 ml/well) to a 24-well plate (referred to as “classic” biofilm) or incubating the inoculated medium in a 50-ml conical tube for 8 h (until early stationary phase) and inverting occasionally (to maintain cells in a planktonic state), followed by transfer to 24-well plate (referred to as “transferred” biofilm). Both types of biofilm were incubated 16 h postinoculation and then washed gently 2 times with PBS, exposed to THY containing various concentrations of penicillin, incubated 1 h, washed again 2 times with PBS, and incubated overnight in fresh THY. Turbidity was then measured using a plate reader at 600 nm, and the lowest concentration of antibiotic giving no turbidity was defined as the MBEC. All incubations were at 37°C.

### Hyaluronidase treatment.

Classic and transferred biofilms were prepared as described for the MBEC assay, except both types of biofilms were incubated for 24 h in 37°C and inoculated 1:100 in medium supplemented with 50 μg/ml hyaluronidase (catalog number H3506; Sigma-Aldrich). Following incubation, biofilm was quantified as described above.

### Surface adhesion.

Fresh THY plus 0.5% glucose was inoculated 1:100 with an overnight culture grown in THY and incubated at 37°C in a conical tube until the desired growth phase was reached: early exponential (OD_600_ of 0.2), late exponential (OD_600_ of 0.8), early stationary (OD_600_ reaching plateau, around 8 h), and late stationary (16 h). Cultures were then spun for 10 min at 8,000 rcf, resuspended, normalized to OD_600_ of 1.0 in PBS, transferred to a 24-well plate (1 ml/well), incubated for 30 min at 37°C, washed twice with PBS, and then stained with 0.1% crystal violet for 10 min and washed again with PBS. Crystal violet was then solubilized in 96% ethanol and quantified at 590 nm using an M200 spectrophotometer (Tecan AG, Männedorf, Switzerland).

### RNA extraction and RT-qPCR.

Bacteria grown in THY plus 0.5% glucose were harvested by centrifugation for 1 min at 10,000 rcf at various growth stages: early exponential (OD_600_ of 0.2), midexponential (OD_600_ of 0.5), late exponential (OD_600_ of 0.8), early stationary (OD_600_ reaching plateau, around 8 h), and late stationary (16 h). RNA was isolated using Direct-zol RNA MiniPrep (Zymo Research, USA) and DNase treated with a Turbo DNA-free kit (Ambion, USA). RNA concentration and DNA absence were assessed by Qubit 2.0 (Invitrogen, USA), and the integrity was determined by TapeStation (Agilent Technologies, USA). Samples with an RNA integrity number (RIN) of >7 and DNA contamination <10% were used for cDNA synthesis using SuperScript III first-strand synthesis kit (Invitrogen). Reverse transcriptase quantitative PCR (RT-qPCR) was performed using a 2× SYBR FAST qPCR universal MasterMix kit (Kappa Biosystems, USA). Gyrase A (*gyrA*) was used as an endogenous control (44). The following primers were used (5′ to 3′): *hasA*, (forward) AGGACGCACTGTCTACCAATC and (reverse) GTCCATAAGGCAACGATGGGA; *gyrA*, (forward) CAACGCACGTAAGGAAGAAA and (reverse) CGCTTGTCAAAACGACGTTA.

### Confocal laser scanning microscopy.

Biofilm in a 3-mm Ibidi dish was washed gently in PBS (twice), fixed with 4% paraformaldehyde for 10 min, washed again with PBS, stained with the dyes indicated below according to the manufacturer’s protocol, and washed again with PBS. Matrix staining was performed using WGA-Alexa Fluor 633 at 50 μg/ml. Hoechst 33342 and Syto9 were used to stain DNA of all cells. Propidium iodide was used to stain DNA of membrane-compromised cells, as it cannot cross the membrane of live cells. For LIVE/DEAD staining, cells were not fixed with paraformaldehyde. Microscopy was performed using a Zeiss LSM780 confocal microscope, equipped with a 20× numerical aperture (NA) 0.80, 63× NA 1.40 (oil), or 100× NA 1.46 (oil) objective lens. Collected Z-stacks were projected as volumes or maximum projections using FIJI distribution of ImageJ (NIH, Bethesda, MD).

### Scanning electron microscopy.

Biofilms in 6-well plates were washed 3 times with 0.1 M phosphate buffer (PB), fixed overnight in 4°C with 2.5% glutaraldehyde (Agar Scientific), postfixed with 1% OsO_4_, dehydrated in an ethanol gradient, and finally dried using hexamethyldisilazane (HMDS; Sigma-Aldrich). After platinum coating, samples were imaged using a Jeol 7610F instrument (Jeol, USA).

## Supplementary Material

Supplemental file 1
