# Supplementary material for: Streptococcus pyogenes Capsule Promotes Microcolony-Independent Biofilm Formation
Source: J Bacteriol. 2019 Aug 22;201(18):e00052-19. doi: 10.1128/JB.00052-19 (PMC6707922; doi:10.1128/JB.00052-19)
Supplement: Supplemental file 1 [file JB.00052-19-s0001.pdf]

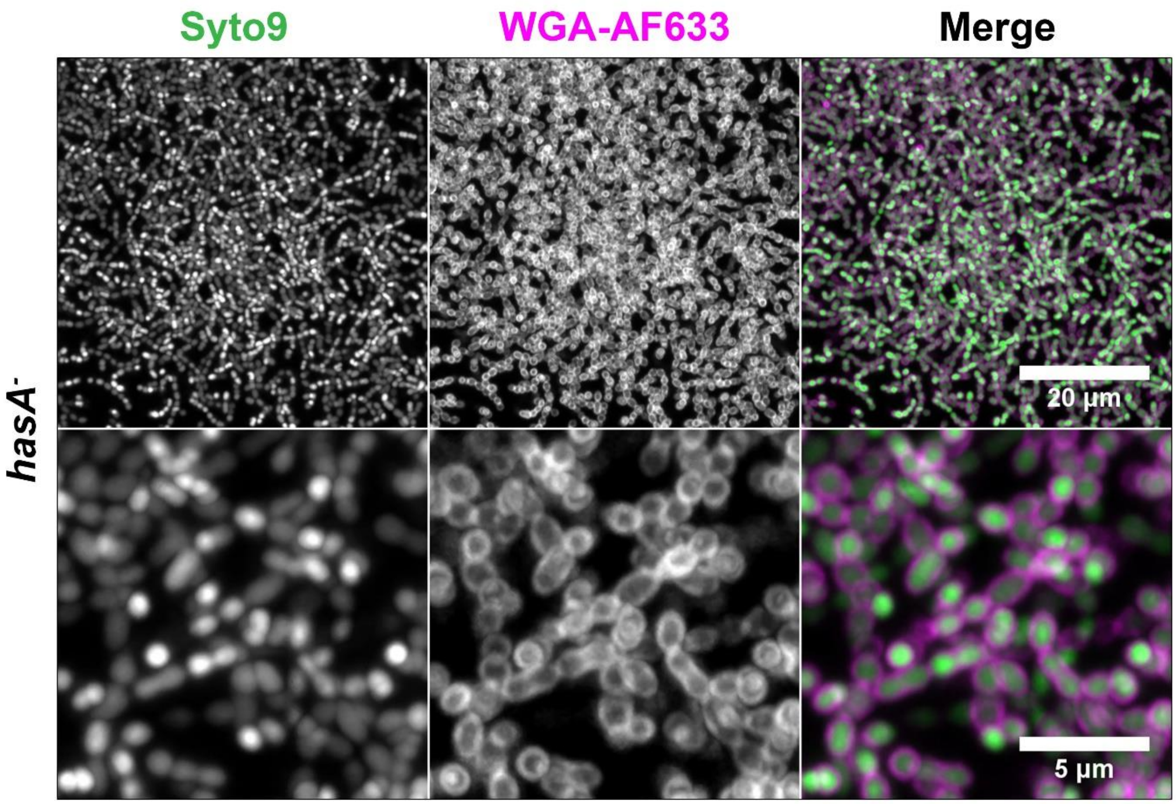

Figure S1. WGA staining. Maximum projection of JS95 *hasA*- biofilm Z-stacks stained with Syto9 (dsDNA) and WGA-Alexa Fluor 633 (carbohydrate/EPS). Positive staining of capsule null mutant confirms that WGA-AF633 signal does not originate from non-specific capsule staining. As in WT, the matrix components visible only at the cell surface but not in extracellular space.

a

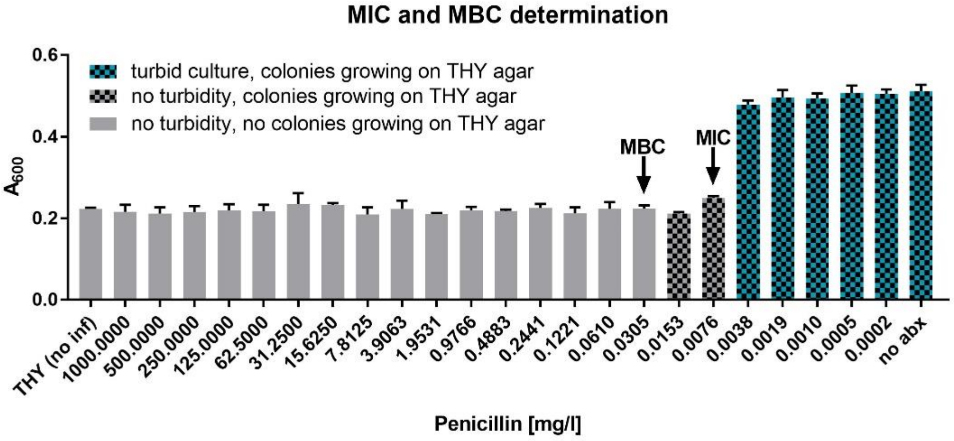

b

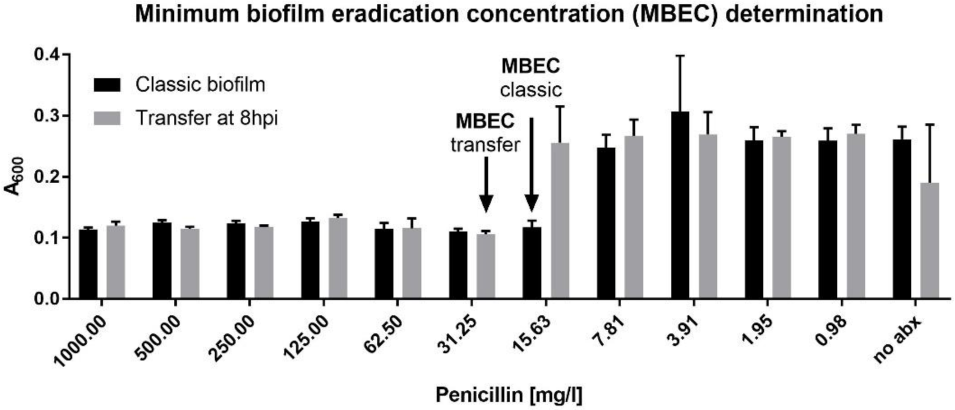

Figure S2. (a) Minimal inhibitory concentration (MIC) and minimal bactericidal concentration (MBC) determination. Bars indicate absorbance at 600nm of cultures incubated overnight with the indicated concentrations of penicillin. Grid patterned bars indicate bacterial growth when plated on THY agar. **(b)** Minimal biofilm eradication concentration (MBEC) estimated by challenging classic and transferred biofilms with penicillin for 1hr, followed by PBS wash and overnight incubation with rescue medium. Bars indicate absorbance measured at 600nm.

a

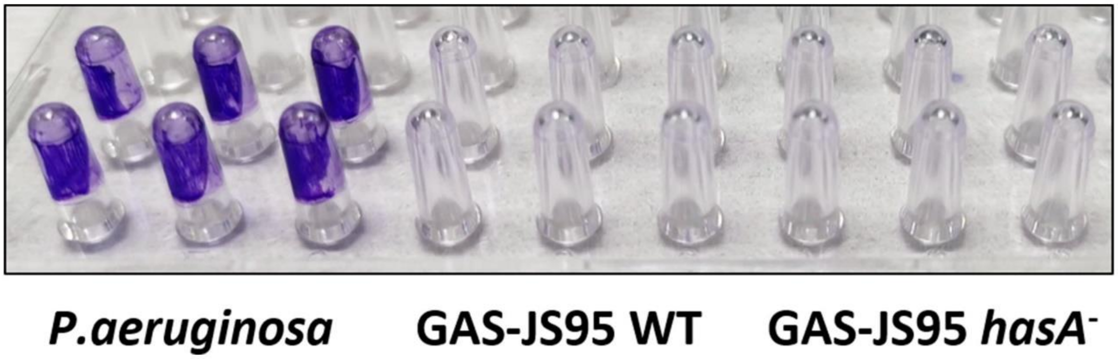

b

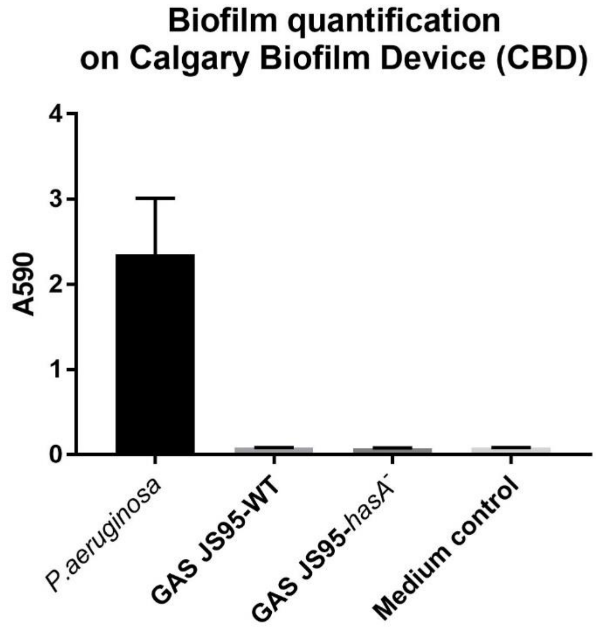

Figure S3. (a) Biofilm grown on pegs of Calgary biofilm device (CBD), washed and stained with crystal violet. *P. aeruginosa*, a classic biofilm former, was used as positive biofilm control. There is no visible growth of JS95 wild type (WT) as well as capsule mutant (*hasA*-) **(b)** Quantification of CBD biofilm after solubilisation in 96% ethanol.
